# Impact of children’s purported past-life memories: a follow-up investigation of American cases

**DOI:** 10.3389/fpsyg.2024.1473340

**Published:** 2024-11-22

**Authors:** Marieta Pehlivanova, Philip J. Cozzolino, Jim B. Tucker

**Affiliations:** Division of Perceptual Studies, Department of Psychiatry and Neurobehavioral Sciences, University of Virginia School of Medicine, Charlottesville, VA, United States

**Keywords:** childhood experiences, absorption, dissociation, fantasy proneness, spirituality, reincarnation

## Abstract

**Introduction:**

Some children between ages 3 and 6 claim to have memories of purported past lives. Prior research has documented this phenomenon in detail, including typical features and how it can manifest in the child’s life. However, less is known about what happens to these children as they transition to adulthood and whether this childhood experience may impact their lives.

**Methods:**

We conducted the first observational follow-up study of American adults (*N* = 23) who were originally interviewed as children regarding their claims of past-life memories. Using online surveys with validated self-report questionnaires and a phone interview, we assessed personality traits, subjective well-being, and the impact of these memories on various domains in their lives.

**Results:**

Adults who reported apparent past-life memories as children seem to lead normal, productive lives and have high educational attainment compared to the general population. They exhibit moderate-to-high levels of spiritual well-being, and slightly elevated, but not pathological, dissociation and fantasy proneness, compared to unselected samples. Sixty-five percent endorsed some impact of the purported memories in their lives, with few reporting negative effects. Spearman’s correlation analysis showed that the degree of impact of this experience was positively associated with the trait of absorption.

**Conclusion:**

This study offers a first glimpse into the lives of American individuals touched by this intriguing childhood experience.

## Introduction

For more than 50 years, researchers have studied the phenomenon of young children reporting purported past-life memories (PLMs).^1^ The vast majority of this work was pioneered and carried out by psychiatrist Ian Stevenson, at the University of Virginia. Stevenson investigated more than one thousand cases and published numerous articles and books presenting detailed case studies and typical features of this phenomenon (Stevenson, 1974; Stevenson, 2001). Most of the early investigations were of Asian cases, in cultures with a prevalent belief in reincarnation, but such cases have been found everywhere researchers have looked. This research still continues today at the University of Virginia, and the primary focus in the past 20 years has been on American cases.

These cases have some common features across cultures. Typically, a child around the age of three, on average, may start talking spontaneously about the purported life of another individual, including how they lived and the manner in which they died (Tucker, 2008; Tucker, 2021). Most children talk about recent lives of ordinary people, usually of the same sex and country. Interestingly, in the majority of cases the life of the other individual ended by unnatural means. Beyond verbal statements, the child may also exhibit behaviors, preferences, phobias or nightmares that appear to be related to the life of the other individual (Tucker, 2008; Stevenson, 1990). Stevenson has argued that this childhood experience may help explain certain aspects of human behavior and identity, including strong likes or dislikes, and gender nonconformity (Tucker, 2008; Stevenson, 1990; Pehlivanova et al., 2018; Stevenson, 1977; Stevenson, 2000), but this perspective remains controversial. Many of these children show strong emotional involvement in the experiences they describe, and some show detachment from their family, which can be distressing for both themselves and their parents (Tucker, 2008).

Most children usually stop talking about the purported life of the other individual by the age of six to seven, as they enter school, and tend to lose the memories of this experience as they get older (Tucker, 2008). The only published data on the prevalence of this phenomenon is a study in Uttar Pradesh, India (Barker and Pasricha, 1979). The authors reported a lower bound on the prevalence of one for every 450 inhabitants. Such cases are likely underreported in the Western world, as parents may dismiss the experience as a transient childhood fantasy or may lack a context in which to interpret it.

Much of the research into the phenomenon of young children’s reports of alleged PLMs has focused on identifying common trends among cases and systematically evaluating various explanations for these experiences. This includes investigating how the child may have acquired information about the life of another individual (usually an adult) and attempting to verify or refute the alleged memories. Even though these PLMs tend to fade or disappear, the experience can have a profound effect on the child’s psyche and behavior while it is active in early childhood (Stevenson, 1990; Stevenson, 2000). However, there has been little research on how this childhood experience may impact individuals’ long-term development into adulthood and whether and how, as adults, they may differ from those who did not have the experience. Independent of research evaluating case-level evidence of the source of children’s claims, this type of follow-up research is important. Parents and families continue to report this unique childhood experience, as suggested by the interest in large social media groups dedicated to providing a safe space for parents to share such stories.^2^ A recent article in a major US newspaper covered the experience of multiple families who have encountered this phenomenon, garnering thousands of reader comments including many from parents sharing similar accounts (Gibson, 2024).

To date, there have only been two published follow-up studies of adults who were originally interviewed as children about their alleged PLMs, both in non-Western countries. The first one was conducted with 42 Sri Lankans of average age 31.5 (range: 19–49) (Haraldsson, 2008). The second study followed up with 27 Lebanese Druze adults of average age 40.3 (range: 28–56), on average 34 years after their initial interviews (Haraldsson and Abu-Izzedin, 2012). According to case study research, in most cases, the experience fades away when the children stop talking about it (Stevenson, 2001). In both follow-up studies, however, a surprisingly high number of individuals who reported spontaneous PLMs as children (75% of Lebanese adults and 38% of Sri Lankan adults) claimed that they had retained some of their alleged memories. Across samples in both countries, a few participants reported that the alleged childhood PLMs had led to unpleasant experiences or difficulties. Among these were instances of unwanted attention and teasing in childhood, as well as phobias which persisted into childhood that participants believed stemmed from their alleged experience as another individual (Haraldsson, 2008; Haraldsson and Abu-Izzedin, 2012). Overall, however, adults in both studies seemed to lead normal, productive lives, and about half of them considered this childhood experience to have had a positive impact on their lives.

This article presents findings from the first exploratory follow-up study of American adults who were first interviewed as children about their alleged PLMs. Given the salience and occasional intrusiveness of these memories in childhood, it is important to understand their long-term effect on the psychosocial development of individuals. This study is an important extension of prior work because it examines the long-term effects of alleged PLMs in a culture without a widespread belief in reincarnation, offering a different context for interpreting this childhood experience. The stigma of the experience in American society may be stronger and there may be an unwillingness to share the experience or to seek help in understanding it (Gibson, 2024). In addition, this population is suitable for investigation as there are no cultural or language barriers, which may have impacted previous follow-up research.

This study used online questionnaires and interviews to assess participant-reported impact of the alleged PLMs. It also assessed established, validated psychological measures, including those specifically associated with reports of various anomalous, if controversial, phenomena. The research on specific personality factors contributing to children’s reports of such experiences is limited. However, we assessed participants’ self-reported levels of absorption (Tellegen and Atkinson, 1974), fantasy proneness (Merckelbach et al., 2001), and dissociation (Bernstein and Putnam, 1986), based on prior research findings. In the context of understanding alleged PLMs, these intercorrelated traits are important to assess because they tend to be elevated among adults who make claims of purported anomalous experiences (Parra, 2006; Cameron and Roll, 1983; Gow et al., 2009). We also administered the Big Five Inventory (John and Srivastava, 1999)—a classic and widely used assessment of the dimensions of human personality. Some of these dimensions have also been linked to reports of and belief in anomalous experiences (Zingrone et al., 1998). Additionally, the measure’s prevalence in the psychological literature allows us to compare our sample to adult reference samples. Based on the published follow-up studies investigating this phenomenon (Haraldsson, 2008; Haraldsson and Abu-Izzedin, 2012), we hypothesized that most of the children went on to have normal adult lives and that the alleged PLMs had no detrimental effect on their lives.

## Method

### Participants and recruitment

The study included 23 American adults who were originally interviewed about their purported PLMs as children by researchers at the University of Virginia (UVA).^3^ The initial pool of potential participants consisted of 124 cases of American children previously studied at UVA who were between ages 18 and 64 at the beginning of the recruitment phase. Every individual who had been interviewed by one of UVA’s investigators about their PLMs was considered. In some cases, the records only included written correspondence, and these individuals were excluded from recruitment (13 cases). Additionally, 19 adults who were never directly interviewed as children, but whose parents were interviewed were not recruited. At least four adults were deceased, and six others could not be located. In all, we attempted to contact 82 adults, of whom 55 simply did not respond, and a few people responded with interest but failed to enroll.

Names, dates and places of birth, former addresses, and familial relationships in the records were used to identify current contact information for potential participants in online databases or services such as Whitepages Premium, ZabaSearch, Facebook, LinkedIn, and Ancestry. In cases of recent contact with the participants, the email address in the files was used to establish contact. Most individuals were otherwise invited to participate in the study via postal mail, and less commonly electronically via social media websites. We made at least one and at most three attempts to contact potential participants who had been identified with a high degree of confidence and who were not deceased according to online records. Some of the letters were returned, and it is not clear whether all unreturned letters reached their intended recipients.

Due to time elapsed between original study participation and the current study, as well as the limited participant pool, recruitment was challenging overall. The long time period made it difficult to track down the current whereabouts of potential participants. Additionally, many children tend to outgrow and stop talking about the PLMs around school age (Tucker, 2008), which could make the original experience less salient.

### Procedure

The study consisted of: (1) an initial 37-item “pre-screening” questionnaire asking about demographics and various aspects of individuals’ PLMs and the effects on their childhood and adult development; (2) a brief phone interview with authors M.P. and J.T., focusing on specific answers provided in the pre-screening questionnaire and other aspects of the case (written notes were taken); (3) an online questionnaire consisting of psychometrically validated self-report measures assessing personality traits, spiritual well-being, and subjective happiness (described below).

Online questionnaires were administered using Qualtrics (Qualtrics, Provo, Utah; site license provided by UVA), via personalized email invitations. The study was approved by UVA’s Institutional Review Board for Social and Behavioral Sciences (protocol # 2601). Participants provided consent electronically in the first online questionnaire. Participants were not paid but were offered a complimentary copy of a book by author J.T. about cases of children reporting PLMs.

### Measures

Participants completed six self-report questionnaires assessing a range of personality traits and well-being. To assess basic dimensions of personality, participants were asked to complete the 44-item Big Five Inventory (BFI) (John and Srivastava, 1999). On this measure, participants are asked to read 44 characteristics “that may or may not apply” to them, using a five-point response scale ranging from (1) *disagree strongly* to (5) *agree strongly*. The scale begins with the stem “I see myself as someone who...” before each characteristic. Example items include (extraversion): “Is talkative”; (agreeableness): “Has a forgiving nature”; (conscientiousness): “Does a thorough job”; (neuroticism): “Is depressed, blue”; (openness): “Is original, comes up with new ideas.”

The 34-item Tellegen Absorption Scale (TAS) was also administered to assess the degree to which participants get mentally absorbed in everyday activities using their imagination (Tellegen and Atkinson, 1974). All items are rated on a binary “true” or “false” response scale. An example item includes, “When I listen to music, I can get so caught up in it that I do not notice anything else.”

Participants also completed the 28-item Dissociative Experiences Scale (DES), which assesses the extent to which individuals lack “the normal integration of thoughts, feelings, and experiences into their stream of consciousness and memory” (Bernstein and Putnam, 1986). Two distinct types of dissociation have been described (Irwin, 1999). Milder nonpathological dissociative experiences can include experiences of psychological absorption, and they are thought to be manifestations of a dissociative trait that occurs on a continuum. More severe experiences, on the other hand, can indicate the potential presence of a dissociative disorder (e.g., dissociative identity disorder). On the DES, participants rate how often they experience 28 examples of daily life occurrences (when they are not under the influence of alcohol or drugs), selecting a percentage between *0% = never* to *100% = always* on a visual analog scale to indicate that item’s frequency. Example items include, “Some people have the experience of driving a car and suddenly realizing that they do not remember what has happened during all or part of the trip” and “Some people sometimes find that they hear voices inside their head that tell them to do things or comment on things that they are doing.”

Participants’ propensity to engage in fantasy was also assessed using the 25-item Creative Experiences Questionnaire (CEQ) (Merckelbach et al., 2001). Participants respond to each item with a *“yes”* or a *“no”* to indicate if the experience described was true or not for them. An example experience includes, “As a child, I thought that the dolls, teddy bears, and stuffed animals I played with were living creatures.”

To assess participants’ self-reported levels of happiness, the four-item Subjective Happiness Scale (SHS) was administered (Lyubomirsky and Lepper, 1999). Items are rated on a 1 to 7 response scale, with different anchors for each item. Examples include, “In general, I consider myself” with the anchors of (1) *not a very happy person* to (7) *a very happy person* and “Compared to most of my peers, I consider myself” with the anchors of (1) *less happy* to (7) *more happy.*

Finally, participants’ well-being related to the spiritual or religious dimensions of life was assessed using the 20-item Spiritual Well-Being Scale (Paloutzian and Ellison, 1982). Participants respond using a scale ranging from (1) *strongly disagree* to (6) *strongly agree*, or (1) *strongly agree* to (6) *strongly disagree* (for negatively-worded items), without a neutral option. Example items from the scale include “I feel a sense of well-being about the direction my life is headed in” and “I believe that God loves me and cares about me.”

### Statistical analysis

Descriptive statistics were summarized as means, medians, and standard deviations for continuous variables and counts and percentages for categorical variables. Due to the small sample, Spearman’s rank correlation coefficients were used to test associations between personality and well-being variables, and degree of impact of the alleged PLMs. To assess the degree of impact of the alleged PLMs, we calculated an impact score based on the total number of questions and domains (out of 7) where participants endorsed any effect, importance, or impact, regardless of its valence or strength. In other words, one point was added to the score for every question where the participant endorsed a non-null response indicating PLM impact. To minimize the likelihood of Type I error in correlations with this degree of impact, a Bonferroni correction controlling for 10 bivariate analyses was used (corrected *p*-value was set at 0.005). The Wilcoxon signed*-*rank test was used to test for differences between average scores on personality traits from the sample and previously reported averages from other reference samples. All statistical analyses were conducted in SAS 9.4.

### Power analysis

Due to the small sample size in this study, we conducted a post-hoc sensitivity power analysis for the correlation analyses, using G*Power Version 3.1.9.7 (Faul et al., 2007). Using an exact two-sided test, a sample of 21 participants (the number for whom we obtained personality data) would be sensitive to effects of *ρ* = 0.57, assuming 80% power, an alpha level of 0.05, and a null hypothesis correlation of 0. In simpler terms, our sample would not reliably detect effects smaller than ρ = 0.57, conventionally a large effect size (Cohen, 2009).

## Results

### Sample characteristics

The sample of adults who were previously interviewed as children about their PLMs was of average age 43.9 ± 13.6 (range: 19–62), and consisted of 13 women (56.5%) and 10 men (43.5%). The average time between the original investigation and the follow-up was 35.8 ± 13.4 years. All participants identified their race as White; additionally, one participant each (4.35%) endorsed the following categories: American Indian or Alaska Native, Asian, and Other (specified as Haitian). Ten participants (43.5%) reported educational attainment of less than a college degree, including high school, some college, or an Associate’s degree. Two participants (8.7%) reported having a high school degree; 8 participants (34.8%) reported educational achievement higher than high school but less than college; 6 participants (26.1%) reported having a college or Bachelor’s degree; and 7 participants (30.4%) reported having completed some graduate studies or earned a Master’s degree. Participants’ occupations spanned a large spectrum of domains, including teaching professionals (e.g., high school teacher, elementary school teacher), health professionals (e.g., physician assistant, registered nurse), other professionals (e.g., architect, air traffic controller, environmental consultant), managers (business executive, higher education administrator), service workers (e.g., donut decorator, childcare worker), machine operators (e.g., forklift operator, operator at a steel mill), and others (e.g., stay-at-home father, unemployed, including due to disability).

Participants were asked to self-report their mental health history, based on a list of psychiatric conditions. Thirty-nine percent said they had been diagnosed with or treated for depression and 30.4% for anxiety. Two participants reported being diagnosed with or treated for obsessive-compulsive disorder and one each for posttraumatic stress disorder, attention-deficit/hyperactivity disorder and autism.

### Persistence, effect, and importance of the childhood experience of alleged PLMs

Most participants (12, 52.2%) stated that all their alleged PLMs had faded away. Although none of the others reported having clear memories of the purported other life they talked about as children, 11 participants (47.8%) said they still had some memories.

### Quantitative data on effects and importance of the childhood experience of alleged PLMs

Table 1 summarizes descriptive data related to the effect, importance, and impact of the alleged PLMs in different domains, based directly on participants’ responses to multiple-choice questions. Parental attitudes toward the memories in childhood were retrospectively perceived as positive by most participants. More than 56% of participants reported that the purported memories had had some importance in their childhood and development. In contrast, only 26% reported that the memories held some importance in their adulthood. Next, participants were asked about the effect of PLMs on specific relationships or aspects of their lives. Nearly three-quarters of participants shared that their alleged PLMs had no effect on their relationships with family members, or with friends and peers. Assessing the aggregate effect, impact, and importance of the experience across different domains and questions, only 35% of participants indicated no effect/impact/importance at all.

**Table 1:** Effect of the alleged memories in different domains, as reported directly by participants.

Questions	Response categories	% (*n*)
Parents’ attitude toward PLMs in childhood	Do not remember	13.0% (3)
	Neutral	0
	Positive	82.6% (19)
	Mixed	0
	Negative	4.3% (1)
Overall effect of PLMs on life and development	No effect	60.9% (14)^1^
	Positive	26.1% (6)
	Mixed effect	8.7% (2)
	Negative	4.3% (1)
Importance of PLMs in childhood and development	Not at all important	43.5% (10)^1^
	Slightly/Moderately important	39.1% (9)
	Very/Extremely important	17.4% (4)
Importance of PLMs in adulthood	Not at all important	73.9% (17)^2^
	Slightly/Moderately important	21.7% (5)
	Very/Extremely important	4.3% (1)
Effect of PLMs on relationships with family members	No effect	73.9% (17)^1^
	Positive	13.0% (3)
	Mixed effect	8.7% (2)
	Negative	4.3% (1)
Effect of PLMs on relationships with friends and peers	No effect	73.9% (17)^1^
	Positive	8.7% (2)
	Mixed effect	8.7% (2)
	Negative	8.7% (2)
Effect of PLMs on romantic relationships	No effect	82.6% (19)^1^
	Positive	4.3% (1)
	Mixed effect	4.3% (1)
	Negative	8.7% (2)
Impact of PLMs on school performance in childhood	No impact	95.7% (22)^1^
	Positive	0
	Mixed impact	0
	Negative	4.3% (1)

^1Includes “I do not remember or I am not sure” category; 2Includes “I am not sure” category.^

### Narrative data on effects and importance of the childhood experience of alleged PLMs

To help contextualize the effect of alleged childhood PLMs (or lack thereof), here we explore narrative responses shared by participants. Some participants who reported that the experience had no effect on their lives spoke matter-of-factly about it.

One 57-year-old woman said:

I did not dwell on the issue as I grew up. I was a child and pushed the memories to the back of my mind and never dwelled on it. My mom did not make a big deal of it and so I did not also. I have gone years at a time without even thinking of it.

Another 57-year-old woman said:

“Past life memories were what they were - a part of me. They did not shape who I am today nor did they shape any of my life choices.”

One recurring theme in multiple responses from participants regarding the positive impacts of alleged PLMs on their lives was the presence of an open-mindedness toward a broader, perhaps more spiritual, view of life and a deepened sense of purpose. A 48-year-old man said:

“The whole idea of it has made me open-minded about a lot of things that some would consider paranormal or whatever. I do not consider these things to be weird.”

Additionally, he expressed a conviction that *“the connections that we have in this lifetime are part of something larger; they do not exist in a vacuum”* and that *“we are all part of something larger.”* A 59-year-old man spoke of how his PLMs inspired a philosophical search for the meaning of life:

Well, knowing that this life that we live is not the only life that you have and that your spirit lives forever, it made me realize that I need to find out what life is all about. How come we are here now and how come we live this life that we are living.

Another prevalent theme among participants’ narrative responses was the influence of alleged childhood PLMs on their views on life and death, particularly in shaping beliefs about the continuation of life after death.

A 26-year-old man shared:

As a child I lost a great grandparent, both grandparents, and a step grandparent. It lessened the blow as I started to understand that they will be back. They may suffer in the end, but there may be a new beginning for them.

Another participant—a 57-year-old woman—shared:

I do not think this is the only life that we have. I believe that people who passed in my life are still around and more than anything it’s affected me to have these people around. These souls or persons do not go away and are still around.

This same participant expressed a desire to talk about her experience with others, yet found it challenging to do so, a sentiment echoed by others. Specifically, she stated *“It’s an integral part of my life, and I still feel that way, as an adult,”* but also expressed that *“society shuns”* the topic.

Very few participants reported that the alleged PLMs had any perceived negative impact on their lives. In one of these examples, a young man had memories of losing a wife whom he could not save in his purported previous life. The participant reported that this fear related to the previous life still carried on in his romantic relationships and he had felt apprehensive about losing his partners.

Among the participants who reported experiencing some intriguing and mixed effects of the alleged PLMs was a 53-year-old woman who was initially interviewed at age four. As a child, she recounted a purported past life as a large, powerful man who got killed by a tiger. As an adult, she recalled that in childhood she was adamant she was not a girl and *“hated being a small, weak girl”* and *“not being in charge.”* In early adolescence, she accepted that she was a girl, which she stated was *“very difficult.”* She emphasized that these gender stereotypes were internal, rather than externally imposed by her family. She noted she was treated the same as her brothers and most women in her family were *“strong.”* However, she felt that she gained great strength from her connection to the man from her childhood experience, which she considered to be positive. The participant had a lifelong interest in hunting, survivalism, and living in the woods—interests which were unusual in her family and which she links to the purported past life.

### Baseline levels of personality and well-being among adults with alleged childhood PLMs

Table 2 shows summary statistics for personality traits and well-being of adults with childhood PLMs, as well as reference averages from previously published data from unrelated adult samples without claimed memories.^4^ All scales or subscales but one showed good to excellent internal consistency, with Kuder–Richardson 20 coefficients of 0.88 for absorption and fantasy proneness and Cronbach’s *α*s in the 0.87–0.95 range for the remaining measures. The only personality measure that achieved adequate but not good internal consistency was the openness subscale of the BFI (Cronbach’s α = 0.64).

**Table 2:** Descriptive statistics of personality traits and well-being among adults who reported alleged PLMs as children, compared to previously published data from unrelated adult samples without reported memories.

Variable	Mean	SD	Median	Range	Cronbach’s Alpha	Reference samples
Absorption	21.1	6.9	21	6–31	0.88^#^	20.3 ± 6 (large sample of undergraduate students) (Glisky et al., 1991)
Dissociation	19.7	13.7	16.8	2.5–51.8	0.91	14.1 ± 13.6 (US adult sample); 18.5 ± 12.9 (US student sample) (Patihis and Lynn, 2017); 23.8 ± 14.1 (US college students); 55.0 ± 19.2 (Dissociative identity disorder patients) (Frischholz et al., 1990)
Fantasy proneness	11.5	6.1	13	0–19	0.88^#^	8.3 ± 3.9 (undergraduates); 9.2 ± 4.4 for men, 8.7 ± 4.0 for women (high school/university students, university employees); 9.5 ± 3.3 (students reporting paranormal experiences); 13.2 ± 4.4 (“fantasy role players”) (Merckelbach et al., 2001)
Subjective happiness	4.8	1.3	4.5	2.5–7	0.86	5.6 ± 1.0 (adult city community sample); 4.9 ± 1.1 (undergraduates at US public university) (Lyubomirsky and Lepper, 1999)
Spiritual well-being*	81.6	25.2	88	42–118	0.95	70.5 ± 17.9 (non-Christian undergraduate students); 82.8 ± 15.0 (Universalists) (Paloutzian and Ellison, 1991)
Agreeableness	3.8	0.9	4	1.3–5	0.88	3.9 ± 0.66 (large sample of adults of age 44) (Srivastava et al., 2003)
Openness	4.0	0.5	4.1	2.9–4.8	0.64	3.9 ± 0.65 (large sample of adults of age 44) (Srivastava et al., 2003)
Neuroticism	3.0	1.1	3.3	1–4.8	0.91	3.1 ± 0.87 (large sample of adults of age 44) (Srivastava et al., 2003)
Extraversion	3.2	1.2	3.3	1.4–4.9	0.93	3.3 ± 0.88 (large sample of adults of age 44) (Srivastava et al., 2003)
Conscientiousness	3.8	0.8	3.9	2–4.8	0.87	3.8 ± 0.70 (large sample of adults of age 44) (Srivastava et al., 2003)

*N* = 21 unless indicated otherwise; *N = 20; SD, standard deviation; ^#^Kuder–Richardson 20 coefficient.

Despite the small sample, the scales exhibited predictable correlation patterns. Absorption, dissociation, and fantasy proneness were all significantly positively associated (ρs: 0.51–0.71; *p*s < 0.02). Spiritual well-being, subjective happiness, and agreeableness were positively associated (ρs: 0.58–0.79; *p*s < 0.007) and all three were negatively associated with neuroticism (ρs: −0.78 – –0.56; *p*s < 0.01).

Absorption in the sample of adults with alleged childhood PLMs was not significantly different from average levels in undergraduate students (*W* = 20.5; *p* = 0.49; Table 2). In terms of dissociation, this sample seemed to show higher average levels than in a US adult sample, although this difference was not significant (*W* = 46.5; *p* = 0.11). However, the average level of dissociation in our sample was lower than one sample of US college students and significantly lower compared to a sample of patients with dissociative identity disorder (DID) (*W* = −115.5; *p* < 0.0001). Although a cutoff of 30 on the DES has been proposed as a criterion with decent sensitivity and specificity in screening (not diagnosing) for DID (Carlson et al., 1993), only 17% of general psychiatric patients scoring 30 or higher would qualify for a DID diagnosis, and this percentage is likely lower in unselected normal populations. In this sample, 4 participants (19% of participants for whom personality data was available) had DES scores of 30 or above. The two largest values (51.4 and 51.8), although not statistical outliers, are both higher than two standard deviations above the average DES. Both participants reported having been diagnosed with or treated for depression and anxiety and one of them additionally reported autism, ADHD, and OCD diagnoses.^5^

Fantasy proneness in this sample was slightly higher than in samples from unselected normal populations (*W*s range: 46.5–58.5; *p*s range: 0.04–0.11), but lower compared to a sample of fantasy role players; however, this difference was not significant (*W* = −38.5; *p* = 0.19). In terms of subjective happiness, the participants were on par with undergraduate students (*W* = −7.5; *p* = 0.80), but scored as less happy compared to a sample of city-dwelling adults (*W* = −67.5; *p* = 0.02).

All 20 participants for whom data was available showed at least moderate levels of spiritual well-being, and 6 (30%) showed high levels (according to respective cutoffs of 41 and 100 outlined in the SWBS scoring manual) (Paloutzian and Ellison, 1991). The SWBS has been used mostly in research with religious/spiritual communities or clinical populations, and not much reference data is available from non-religious healthy participants. Nonetheless, adults with alleged childhood PLMs appeared to show higher spiritual well-being than non-Christian undergraduate students, but this difference was not significant (*W* = 44.5; *p* = 0.10). However, they showed similar levels to Unitarians (*W* = −6; *p* = 0.83)—an inclusive spiritual belief system drawing from both Eastern and Western religions and philosophies.

Lastly, all BFI personality traits in this sample closely matched averages in a large sample of similar age (*W*s range: 4.5–40; *p*s range: 0.14–0.88).

### Association between degree of impact of alleged childhood PLMs and personality and well-being variables

The degree of impact of the PLMs was assessed as a count of questions and domains for which adults endorsed some effect/importance/impact (out of 7, presented in Table 1). Descriptive statistics for this PLM impact score are presented in Table 3. Sixty-five percent of participants endorsed some impact of the alleged PLMs in their lives. Associations between degree of impact of the alleged PLMs and personality and well-being variables are presented in Table 4. Absorption showed a moderate to strong positive correlation with impact scores, while openness to experience showed a moderate positive correlation. After adjusting for multiple testing, only the absorption effect remained significant.

**Table 3:** Degree of impact of alleged PLMs as measured by number of questions for which participants endorsed any effect/importance/impact of the PLMs (out of 7).

Impact score	% (*n*)
0	34.5% (8)
1	13.0% (3)
2	17.4% (4)
3	13.0% (3)
4	4.4% (1)
5	13.0% (3)
6	4.4% (1)
7	0% (0)

*N = 23.*

**Table 4:** Spearman correlations between degree of impact of alleged PLMs and personality and well-being variables.

Variable	Spearman’s *ρ*	*p*-value
Absorption	0.60	.005^ⱡ+^
Dissociation	0.37	0.10
Fantasy proneness	0.35	0.12
Subjective happiness	−0.03	0.88
Spiritual well-being*	−0.05	0.84
Agreeableness	−0.20	0.39
Openness	0.44	0.05^
Neuroticism	0.03	0.91
Extraversion	−0.06	0.79
Conscientiousness	−0.34	0.13

*N* = 21, unless otherwise specified. *N = 20; ⱡ *p*-value less than 0.005 before rounding; + significant after Bonferroni correction; ^ *p*-value less than 0.05 before rounding.

### Effect on belief in reincarnation

Due to the interpretation by some children that they may have lived a previous life, we asked participants about their current belief regarding reincarnation. The majority (65.2%, 15) of participants endorsed a belief in reincarnation, while an equal percentage (17.4%, 4) either reported being uncertain or responded negatively. Notably, these rates of endorsement of reincarnation are considerably higher than the 27% of Americans who hold such beliefs, as reported by Pew Research Center (2023).

## Discussion

This study presents a follow-up assessment of American adults who were interviewed as children about their alleged past-life memories. Results indicate that the adults who expressed these memories as children lead normal, productive lives with families, relationships, and jobs across the spectrum of responsibilities and incomes. Among this sample of adults, there was very little evidence of self-reported detrimental impact of this experience in adulthood. Their educational attainment was high, with 13 of the 23 reporting either undergraduate or graduate degrees. Overall, these findings are consistent with the previous follow-up studies in Lebanon and Sri Lanka that reported that adults with alleged childhood PLMs led normal lives (Haraldsson, 2008; Haraldsson and Abu-Izzedin, 2012). Additionally, participants in the Sri Lankan study reported higher educational attainment compared to peers (Haraldsson, 2008), consistent with our findings in the current sample.

On average, the participants of the current study did not report major detrimental effects that they attribute to this experience. The rate of reported negative impacts in this sample was slightly lower than that observed in the Lebanese sample (Haraldsson and Abu-Izzedin, 2012), and considerably lower than in the Sri Lankan sample (Haraldsson, 2008). The rate of self-reported remembrance of the alleged PLMs in this American sample was significantly lower than among Lebanese adults (Haraldsson and Abu-Izzedin, 2012), and was comparable but slightly lower than the rate reported among Sri Lankan adults (Haraldsson, 2008).

The finding that children who express purported PLMs grow up to become functional, productive adults is consistent with studies that have assessed psychological functioning at earlier ages. Tucker and Nidiffer conducted a psychological evaluation and cognitive testing of 15 American children, ages 3–6, who had made repeated statements about alleged PLMs (Tucker and Nidiffer, 2014). These children scored above average in terms of intelligence and adaptive behaviors. Data comparing children with purported PLMs from Sri Lanka to control children without memories further corroborate these findings (Haraldsson, 1997). The children who had made purported past-life reports had greater verbal skills and intelligence, as well as better memory, and they performed much better in school and were not more suggestible than the controls.

### Psychological profile of American adults with alleged childhood PLMs

On average, American adults who expressed purported PLMs as children had similar Big Five and absorption personality patterns compared to unselected samples. However, on average, they showed elevated, but non-pathological, levels of dissociation and fantasy proneness. In the study evaluating American children while they are actively reporting the memories, Tucker and Nidiffer reported low levels of dissociation in this group (Tucker and Nidiffer, 2014). However, studies with Sri Lankan (Haraldsson et al., 2000) and Lebanese (Haraldsson, 2003) children have similarly found elevated, but non-pathological, dissociation scores among children with purported PLMs compared to children with no memories. Haraldsson also reported slightly elevated levels of fantasy proneness among Lebanese children when investigating this phenomenon (Haraldsson, 2003).

Specifically, two participants in the current study showed high scores on the Dissociative Experiences Scale. Nonetheless, there is not a clear link between these elevated scores and the memories in this sample of adults. Both adults with DES scores higher than 50 were studied as children in Tucker and Nidiffer’s psychological evaluation (Tucker and Nidiffer, 2014), including an assessment on the Child Dissociative Checklist (Putnam et al., 1993). Both participants had very low scores, indicating no dissociative tendencies at that time. Moreover, these two adults are among the 52% of the sample who say the alleged memories they once expressed are no longer accessible. Decades of research indicates that the typical features observed in cases of alleged childhood PLMs do not match the classic presentation of pathological dissociation (Stevenson, 2001; Tucker, 2008; Tucker, 2021), as assessed by the DES-Taxon (Waller and Ross, 1997). For example, when talking about the alleged past life, children do not claim to “experience themselves in another place,” nor do they fail to recognize their current family members, nor do they feel like “people, objects and the world around them are not real,” to name only a few examples from the subscale. Nonetheless, there is reason to continue assessing dissociation as a co-occurring outcome among children who report alleged PLMs, as a potentially relevant factor in a subset of these cases. However, these data, in conjunction with previous research (Tucker and Nidiffer, 2014), suggest that the connection between this childhood experience and dissociation may not be causal in nature.

Overall, adults in this sample showed moderate to high self-reported spiritual well-being. The study did not directly test the link between spiritual well-being and the impact of the alleged memories. However, several participants reported some positive spiritual impacts that they attributed to the experience, including a deepened sense of purpose, and expanded and adaptive views on life and death. This suggests the possibility that this childhood experience may have a positive effect on adult spiritual well-being. Even though participants showed average scores on absorption and Big Five personality traits, there were some interesting associations between some of these traits and the degree of impact of the memories, which are discussed in the following section.

Despite the high spiritual well-being scores, the self-reported levels of diagnosed or treated depression or anxiety appear to be high among this group (39.1 and 30.4%, respectively). Nonetheless, these self-reported rates are largely consistent with research exploring recent data from the US Census Bureau’s Household Pulse Survey (Panchal et al., 2023). That study found that 32.3% of all American adults reported symptoms of anxiety and/or a depressive disorder, and that percentage increased to 44% among 18-to 49-year-olds. Additionally, according to a recent Pew Research Center report on mental health during the COVID-19 pandemic, which overlaps with some of the data collection for this study, 41% of American adults self-reported high levels of psychological distress (Pasquini and Keeter, 2022).

### Effect of children’s alleged past-life memories

The results section on how this unique childhood experience can impact the children as they age highlighted the experience of a woman who had purported memories of another life as a man. Her experience and the behavioral and attitudinal preferences she attributed to the purported past life align with a previously reported finding in cases where children express memories of being a different gender. Based on a large database of cases of children’s alleged PLMs, children claiming a life of a different gender are much more likely to exhibit gender nonconformity than those reporting a purported past life of the same gender (Pehlivanova et al., 2018). This participant’s account, gathered nearly 50 years after her initial childhood interview, provides an intriguing perspective on the trajectory of alleged PLMs in conjunction with gender nonconformity. The impacts of this experience in this particular context should be explored more deeply in a future study following up on cases of children reporting alleged memories of being a different gender.

Certain aspects of personality showed associations with the degree of lifelong impact of this childhood experience in participants’ lives. Despite average levels of absorption in the sample overall, adults who scored higher on absorption reported a significantly stronger impact of the alleged memories. Absorption as a psychological trait measures individuals’ tendency to become deeply absorbed and immersed in experiences, which could include mental imagery or sensory experiences (Tellegen and Atkinson, 1974; Roche and McConkey, 1990). Absorption, along with fantasy proneness and dissociation, is part of a cluster of intercorrelated personality traits that have been shown to correlate with a range of anomalous and extraordinary experiences (Parra, 2006; Gow et al., 2009; Acunzo et al., 2020). Drawing from diverse bodies of research (including on religious, psychedelic, and psychic experiences), Lifshitz and colleagues suggest that absorption underlies the general tendency to have spiritual experiences, by allowing individuals to “to experience something immaterial as present and real” (Lifshitz et al., 2019). (They note that they are not necessarily implying that the immaterial or spiritual world is not real, but rather that high absorption may allow individuals to directly experience it.)

Due to the correlational nature of the current study, the direction of causality in the association between absorption and degree of impact of alleged PLMs cannot be definitively determined. However, given that absorption seems to predispose individuals to extraordinary or spiritual experiences, it is plausible that higher absorption among some individuals who had this childhood experience may facilitate the ongoing connection to and engagement with it as their life progresses. Consistent with the mechanisms discussed by Lifshitz and colleagues, one possible pathway is that absorption genuinely increases the likelihood that the alleged PLMs will impact some individuals (Lifshitz et al., 2019). It is also possible that absorption instead or in addition makes some individuals more likely to attribute aspects of their life and development to this childhood experience.

In addition to absorption, the trait of openness to experience was also positively associated with degree of impact of the alleged PLMs, but this effect did not survive corrections for multiple testing. Nonetheless, this pattern of findings is consistent, as absorption and openness are conceptually related and consistently positively correlated (Glisky et al., 1991; Radtke and Stam, 1991), and high openness is also associated with various psychic experiences (Zingrone et al., 1998). Openness to experience is a multifactorial dimension of personality, and absorption is “most closely associated with subscales. … related to … unusual perceptions and associations, fantasy and dreams, unconventional views of reality, and awareness of inner feelings” (Glisky et al., 1991). Similarly to the absorption pathways outlined above, these aspects of openness can predispose individuals to be impacted by this childhood experience and/or to draw connections between the experience and other developments in their lives. Fantasy proneness and dissociation—other personality traits related to absorption as well as purported psychic experiences—showed correlations in the anticipated direction. We speculate that these effects might well have been significant in a larger sample with greater statistical power.

## Limitations

The main limitation of this study is the relatively small sample size, reducing statistical power and the generalizability of the findings. Additionally, the sample of adults who were interviewed about their alleged PLMs as children may not be representative of all such cases in the research collection at UVA or in general of adults who experienced this phenomenon as children. It is plausible that adults who had remaining memories of this childhood experience or a positive experience with either the memories or the original research study were more likely to participate than those who did not. Despite these limitations, studying a unique but small pool of individuals who reported alleged childhood PLMs offers novel insights into the experience of these children as they develop into adulthood.

## Future directions

Following up with American adults who had participated in a research study with the University of Virginia 36 years earlier, on average, represents a significant new direction in the investigation of children’s purported PLMs. Moving forward, it will be important to conduct longitudinal prospective investigations, assessing children and their families at more frequent intervals. This longitudinal approach would allow for a more refined assessment of the social and psychological consequences or correlates of this childhood experience (including death attitudes, gender nonconformity, or phobias and philias). Furthermore, it would enable researchers to trace the trajectories of important outcomes for both the children and their parents to assess whether and how they diverge or converge. Also, regardless of its etiology, a child’s claim to remember a past life—often involving an unnatural or even violent death—comes with a set of unique challenges for the children and their families. It is important to assess these challenges in a detailed and continuing manner. Finally, future follow-up investigations should include assessments of psychopathology (including potentially pathological dissociation), attachment styles, social development, and interpersonal relationship quality.

## Conclusion

The phenomenon of children reporting purported memories of past lives has been investigated for decades by psychiatrists and other researchers, with a recent focus on American cases (Stevenson, 1974; Stevenson, 2001; Tucker, 2021). Most of this work has focused on investigating these experiences while they are occurring in childhood, but less research has been devoted to following up with these children in adulthood. The findings from this study—the first involving American adults who were once investigated as children regarding their claimed past-life memories—align with earlier studies from other cultures, demonstrating that these children grow up to lead normal lives as adults. Even though this phenomenon appears to be rare and may not be fully understood, it is important to keep studying it and to normalize it as a childhood experience that does not seem to leave a negative mark on individuals. Broadly, this research has implications for understanding early childhood and life consequences that may stem from it, as well as for characterizing and understanding extraordinary and anomalous human experiences.

## Data Availability

The dataset presented in this article are not readily available because quantitative data from this study and the code used to analyze it will be made available by request to the corresponding author from qualified researchers who express interest in reviewing them. Requests to access the dataset and analysis code should be directed to Marieta Pehlivanova, mp8ce@uvahealth.org.

## Footnotes

^1^Purported past-life memories will occasionally be referred to as PLMs or memories, with no claims of veracity. Many researchers contributing to the academic literature on this phenomenon interpret the children’s statements and behaviors, in some cases, to bear strong correspondence to the known life of another deceased individual. However, this study and article are strictly concerned with the impact of this childhood experience on individuals and remain agnostic to any interpretation that the memories claimed by these children may be “real.” Importantly, we are specifically referring to the childhood experience of some young children reporting “memories” of purported past lives spontaneously, rather than adults’ claims of acquiring such "memories" during dreams, meditation, or hypnosis.

^2^For one example, the public Facebook group titled “Verifiable Children’s Reincarnation Stories” has nearly 230 K members and hundreds of posts each month. Link: https://www.facebook.com/groups/vcrstories.

^3^Twenty-nine parents of investigated children with PLMs were also enrolled, and parent data will be reported separately.

^4^In this study, we did not have access to a demographically matched control group of adults confirmed not to have had alleged childhood PLMs. In the absence of such a control group, we sought to identify the most relevant adult reference samples for each variable, acknowledging the limitations of the available published literature.

^5^Among the four participants scoring high on the DES measure, we calculated a specific subscore (the DES-Taxon) from the full scale, which more accurately classifies cases of pathological dissociation (Waller and Ross, 1997). Of the four participants scoring high on the full measure, only two—the same two who scored the highest on the full measure—also obtained DES-Taxon scores above 30. Among individuals scoring 30 or higher on the DES-Taxon, 87% will have an actual dissociative disorder (Waller and Ross, 1997).

## References

[ref9001] Acunzo D. Cardeña E. Terhune D. B. (2020). Anomalous experiences are more prevalent among highly suggestible individuals who are also highly dissociative. Cogn. Neuropsychiatry. 25:179–189., PMID: 31955650 10.1080/13546805.2020.1715932

[ref1] Barker D. R. Pasricha S. K. (1979). Reincarnation cases in Fatehabad: a systematic survey in North India. J. Asian Afr. Stud. 14, 231–240. doi: 10.1177/002190967901400304

[ref2] Bernstein E. M. Putnam F. W. (1986). Development, reliability, and validity of a dissociation scale. J. Nerv. Ment. Dis. 174, 727–735. doi: 10.1097/00005053-198612000-00004 3783140

[ref3] Cameron T. Roll W. G. (1983). An investigation of apparitional experiences. Theta 11, 74–78.

[ref4] Carlson E. B. Putnam F. W. Ross C. A. Torem M. Coons P. Dill D. L.. (1993). Validity of the dissociative experiences scale in screening for multiple personality disorder: a multicenter study. Am. J. Psychiatry 150, 1030–1036. doi: 10.1176/ajp.150.7.1030, PMID: 8317572

[ref5] Cohen J. (2009). Statistical power analysis for the behavioral sciences. 2nd Edn. New York, NY: Psychology Press, 567.

[ref6] Faul F. Erdfelder E. Lang A. G. Buchner A. (2007). G*power 3: a flexible statistical power analysis program for the social, behavioral, and biomedical sciences. Behav. Res. Methods 39, 175–191. doi: 10.3758/BF03193146, PMID: 17695343

[ref7] Frischholz E. J. Braun B. G. Sachs R. G. Hopkins L.. (1990). The dissociative experiences scale: further replication and validation. Dissociation Prog Dissociative Disord. 3, 151–153.

[ref8] Gibson C. The children who remember their past lives. Washington Post. (2024) Available at: https://www.washingtonpost.com/lifestyle/2024/05/02/children-past-lives/ (Accessed February 10, 2024).

[ref9] Glisky M. L. Tataryn D. J. Tobias B. A. Kihlstrom J. F. McConkey K. M. (1991). Absorption, openness to experience, and hypnotizability. J. Pers. Soc. Psychol. 60, 263–272. doi: 10.1037/0022-3514.60.2.263, PMID: 2016669

[ref10] Gow K. M. Hutchinson L. Chant D. (2009). Correlations between fantasy proneness, dissociation, personality factors and paranormal beliefs in experiencers of paranormal and anomalous phenomena. Aust. J. Clin. Exp. Hypn. 37, 169–191.

[ref11] Haraldsson E. (1997). A psychological comparison between ordinary children and those who claim previous-life memories. J. Sci. Explor. 11, 323–335.

[ref12] Haraldsson E. (2003). Children who speak of past-life experiences: is there a psychological explanation? Psychol. Psychother. Theory Res. Pract. 76, 55–67. doi: 10.1348/14760830260569256 12689435

[ref13] Haraldsson E. (2008). Persistence of past-life memories: study of adults who claimed in their childhood to remember a past life. J. Sci. Explor. 22, 385–393.

[ref14] Haraldsson E. Abu-Izzedin M. (2012). Persistence of “past-life” memories in adults who, in their childhood, claimed memories of a past life. J. Nerv. Ment. Dis. 200, 985–989. doi: 10.1097/NMD.0b013e3182718c51, PMID: 23124184

[ref15] Haraldsson E. Fowler P. C. Periyannanpillai V. (2000). Psychological characteristics of children who speak of a previous life: a further field study in Sri Lanka. Transcult. Psychiatry 37, 525–544. doi: 10.1177/136346150003700403

[ref16] Irwin H. J. (1999). Pathological and nonpathological dissociation: the relevance of childhood trauma. J. Psychol. 133, 157–164. doi: 10.1080/00223989909599730, PMID: 10188264

[ref17] John O. P. Srivastava S. (1999). “The big five trait taxonomy: history, measurement, and theoretical perspectives” in Handbook of personality: Theory and research. eds. Pervin L. O. John O. P.. 2nd ed (New York, NY, US: Guilford Press), 102–138.

[ref18] Lifshitz M. van Elk M. Luhrmann T. M. (2019). Absorption and spiritual experience: a review of evidence and potential mechanisms. Conscious. Cogn. 73:102760. doi: 10.1016/j.concog.2019.05.008, PMID: 31228696

[ref19] Lyubomirsky S. Lepper H. S. (1999). A measure of subjective happiness: preliminary reliability and construct validation. Soc. Indic. Res. 46, 137–155. doi: 10.1023/A:1006824100041

[ref20] Merckelbach H. Horselenberg R. Muris P. (2001). The creative experiences questionnaire (CEQ): a brief self-report measure of fantasy proneness. Personal. Individ. Differ. 31, 987–995. doi: 10.1016/S0191-8869(00)00201-4

[ref21] Paloutzian R. F. Ellison C. W. (1982). “Loneliness, spiritual well-being and the quality of life” in Loneliness: a sourcebook of current theory, research and therapy. eds. Peplau L. A. Perlman D. (New York: Wiley), 224–237.

[ref22] Paloutzian R. F. Ellison C. W. (1991). Manual for the spiritual well-being scale. Nyack, New York: Life Advance.

[ref23] Panchal N Saunders H Rudowitz R Cox Cynthia. (2023). The implications of COVID-19 for mental health and substance use. Available at: https://www.kff.org/mental-health/issue-brief/the-implications-of-covid-19-for-mental-health-and-substance-use/ (Accessed 29 May, 2024).

[ref24] Parra A. (2006). Seeing and feeling ghosts: absorption, fantasy proneness, and healthy schizotypy as predictors of crisis apparition experiences. J. Parapsychol. 70, 357–372.

[ref25] Pasquini G Keeter S. At least four-in-ten U.S. adults have faced high levels of psychological distress during COVID-19 pandemic. Pew Research Center. (2022). Available at: https://www.pewresearch.org/short-reads/2022/12/12/at-least-four-in-ten-u-s-adults-have-faced-high-levels-of-psychological-distress-during-covid-19-pandemic/ (Accessed May 29, 2024).

[ref26] Patihis L. Lynn S. J. (2017). Psychometric comparison of dissociative experiences scales II and C: a weak trauma-dissociation link. Appl. Cogn. Psychol. 31, 392–403. doi: 10.1002/acp.3337

[ref27] Pehlivanova M. Janke M. J. Lee J. Tucker J. B. (2018). Childhood gender nonconformity and children’s past-life memories. Int. J. Sex. Health 30, 380–389. doi: 10.1080/19317611.2018.1523266

[ref28] Pew Research Center. Spirituality among Americans. (2023) Available at: https://www.pewresearch.org/religion/2023/12/07/spirituality-among-americans/ (Accessed July 12, 2023).

[ref29] Putnam F. W. Helmers K. Trickett P. K. (1993). Development, reliability, and validity of a child dissociation scale. Child Abuse Negl. 17, 731–741. doi: 10.1016/S0145-2134(08)80004-X, PMID: 8287286

[ref30] Radtke H. L. Stam H. J. (1991). The relationship between absorption, openness to experience, anhedonia, and susceptibility. Int. J. Clin. Exp. Hypn. 39, 39–56. doi: 10.1080/00207149108409617, PMID: 2001896

[ref31] Roche S. M. McConkey K. M. (1990). Absorption: nature, assessment, and correlates. J. Pers. Soc. Psychol. 59, 91–101. doi: 10.1037/0022-3514.59.1.91

[ref32] Srivastava S. John O. P. Gosling S. D. Potter J. (2003). Development of personality in early and middle adulthood: set like plaster or persistent change? J. Pers. Soc. Psychol. 84, 1041–1053. doi: 10.1037/0022-3514.84.5.1041 12757147

[ref33] Stevenson I. (1974) Twenty Cases Suggestive of Reincarnation (Rev. ed.). Charlottesville: University Press of Virginia.

[ref34] Stevenson I. (1977). The southeast Asian interpretation of gender dysphoria: an illustrative case report. J. Nerv. Ment. Dis. 165, 201–208. doi: 10.1097/00005053-197709000-00008, PMID: 142818

[ref35] Stevenson I. (1990). Phobias in children who claim to remember previous lives. J Sci Explor. 4, 243–254. doi: 10.1016/j.explore.2024.103063

[ref36] Stevenson I. (2000). The phenomenon of claimed memories of previous lives: possible interpretations and importance. Med. Hypotheses 54, 652–659. doi: 10.1054/mehy.1999.0920, PMID: 10859660

[ref37] Stevenson I. (2001). Children who remember previous lives a question of reincarnation. Revised Edn. Jefferson, NC: McFarland.

[ref38] Tellegen A. Atkinson G. (1974). Openness to absorbing and self-altering experiences (“absorption”), a trait related to hypnotic susceptibility. J. Abnorm. Psychol. 83, 268–277. doi: 10.1037/h0036681, PMID: 4844914

[ref39] Tucker J. B. (2008). Children’s reports of past-life memories: a review. Explore 4, 244–248. doi: 10.1016/j.explore.2008.04.001, PMID: 18602617

[ref40] Tucker J. B. (2021). “Cases of the reincarnation type” in Consciousness unbound liberating the mind from the tyranny of materialism. eds. Kelly E. F. Marshall P. (Rowman & Littlefield), 57–87.

[ref41] Tucker J. B. (2021). Before: Children’s memories of previous lives. New York: St. Martin’s Essentials, 544.

[ref42] Tucker J. B. Nidiffer F. D. (2014). Psychological evaluation of American children who report memories of previous lives. J Sci Explor. 28, 583–594.

[ref43] Waller N. G. Ross C. A. (1997). The prevalence and biometric structure of pathological dissociation in the general population: taxometric and behavior genetic findings. J. Abnorm. Psychol. 106, 499–510. doi: 10.1037/0021-843X.106.4.499, PMID: 9358680

[ref44] Zingrone N. L. Alvarado C. S. Dalton K. (1998). Psi experiences and the “big five”: relating the NEO-PI-R to the experience claims of experimental subjects. Eur. J. Parapsychol. 14, 31–51.

